# Autotaxin is induced by TSA through HDAC3 and HDAC7 inhibition and antagonizes the TSA-induced cell apoptosis

**DOI:** 10.1186/1476-4598-10-18

**Published:** 2011-02-12

**Authors:** Song Li, Baolu Wang, Yan Xu, Junjie Zhang

**Affiliations:** 1 The Key Laboratory for Cell Proliferation and Regulation Biology of Ministry of Education, College of Life Sciences, Beijing Normal University, Beijing 100875, China; 2 Department of Obstetrics and Gynecology, Indiana University Cancer Center, Indiana University School of Medicine, 975 West Walnut Street, IB355A, Indianapolis, IN 46202, USA

## Abstract

**Background:**

Autotaxin (ATX) is a secreted glycoprotein with the lysophospholipase D (lysoPLD) activity to convert lysophosphatidylcholine (LPC) into lysophosphatidic acid (LPA), a bioactive lysophospholipid involved in diverse biological actions. ATX is highly expressed in some cancer cells and contributes to their tumorigenesis, invasion, and metastases, while in other cancer cells ATX is silenced or expressed at low level. The mechanism of ATX expression regulation in cancer cells remains largely unknown.

**Results:**

In the present study, we demonstrated that trichostatin A (TSA), a well-known HDAC inhibitor (HDACi), significantly induced ATX expression in SW480 and several other cancer cells with low or undetectable endogenous ATX expression. ATX induction could be observed when HDAC3 and HDAC7 were down-regulated by their siRNAs. It was found that HDAC7 expression levels were low in the cancer cells with high endogenous ATX expression. Exogenous over-expression of HDAC7 inhibited ATX expression in these cells in a HDAC3-dependent manner. These data indicate that HDAC3 and HDAC7 collaboratively suppress ATX expression in cancer cells, and suggest that TSA induce ATX expression by inhibiting HDAC3 and HDAC7. The biological significance of this regulation mechanism was revealed by demonstrating that TSA-induced ATX protected cancer cells against TSA-induced apoptosis by producing LPA through its lysoPLD activity, which could be reversed by BrP-LPA and S32826, the inhibitors of the ATX-LPA axis.

**Conclusions:**

We have demonstrated that ATX expression is repressed by HDAC3 and HDAC7 in cancer cells. During TSA treatment, ATX is induced due to the HDAC3 and HDAC7 inhibition and functionally antagonizes the TSA-induced apoptosis. These results reveal an internal HDACi-resistant mechanism in cancer cells, and suggest that the inhibition of ATX-LPA axis would be helpful to improve the efficacy of HDACi-based therapeutics against cancer.

## Introduction

Autotaxin (ATX), also known as nucleotide pyrophosphatase/phosphodiesterase 2 (NPP2), is an exo-enzyme originally identified as a tumor cell autocrine motility factor [1]. Different from NPP1 and NPP3, ATX is synthesized as a pre-pro-enzyme, and the removal of the propeptide by furin-like proteases is required for its full activation [2]. ATX is present in most biological fluids, including cerebro-spinal fluid, plasma, peritoneal fluid, urine, and synovial fluid [3], with the lysophospholipase D (lysoPLD) activity converting lysophosphatidylcholine (LPC) into lysophosphatidic acid (LPA) [4]. It has been reported that ATX is rapidly cleansed from the circulation by liver sinusoidal endothelial cells (LSECs) [5]. ATX deficiency in mouse leads to embryonic lethality, indicating that ATX is required for normal development. Compared with the wild-type mice, ATX heterozygous mice develop normally, but have half plasma LPA levels [6,7]. Therefore, ATX is regarded as a major enzyme to produce LPA in the blood and potentially other biological fluids. Many, if not all, biological functions of ATX appear to be mediated by LPA signaling. LPA acts on specific G protein-coupled receptors to regulate a wide range of cellular activities, ranging from cell proliferation, differentiation, migration, to anti-apoptosis [8]. To date, at least six LPA receptors have been identified and additional unidentified LPA receptors may still exist [9]. The best-known LPA receptors are LPA_1_, LPA_2_, and LPA_3_, which are members of the endothelial differentiation gene (EDG) family [9]. The broad range of LPA cellular functions is accomplished by the different LPA receptors differentially coupled to distinct G proteins (Gq, Gi and G12/13) and their down-stream signaling molecules, including phospholipase C, PI3K, Ras-MAPK, Rac, and Rho [10].

ATX plays roles in the immune [11] and the nervous systems [12], as well as in angiogenesis [13,14]. In addition, the significant functions of the ATX-LPA axis have been demonstrated in several cancer types. Autotaxin (ATX), which was initially isolated as a prometastatic enzyme from the conditional medium of human melanoma cells [1], is over-expressed in several human cancers and contributes to their progression, such as non-small cell lung cancer, breast cancer, renal cell cancer, prostate cancer, hepatocellular carcinoma, thyroid cancer and neuroblastoma [15]. Ectopic expression of ATX in ras-transformed NIH3T3 cells stimulates their tumorigenesis and metastatic potential [16]. ATX largely accounts for the motility of MDA-MB-435 cells [17], and the expression of ATX and lysophosphatidic acid receptors increases mammary tumorigenesis [18]. ATX-LPA axis also facilitates cancer cells survival under drug treatment. It has been reported that ATX protects MDA-MB-435 cells against taxol-induced apoptosis and delays apoptosis induced by carboplatin in OVCAR-3 ovarian cancer cells through LPA generation [19,20]. Therefore, ATX is regarded as an attractive target of cancer therapy [21].

ATX expression is inducible by VEGF, EGF, bFGF and BMP-2, but inhibited by TGF-β and several cytokines, including IL-1, IL-4 and IFN-γ [15]. We have recently reported that ATX expression is regulated by TNF-α in human hepatocellular carcinoma [22]. However, most of these regulations are cell type- and/or context-specific. The endogenous ATX expression is high in some cancer cells, but low or undetectable in other cancer cell types [23]. Hence, the mechanisms by which endogenous ATX expression is regulated in cancer cells remain to be further explored.

Histone deacetylases (HDACs) comprise of a family of 18 genes, which are grouped into classes I-IV based on their homology to their respective yeast orthologues [24]. In addition to histone proteins, HDACs have many non-histone protein substrates which play roles in gene expression regulation [25]. HDACs are involved in various cellular processes, such as DNA replication, cell cycle progression, gene silencing, cell differentiation and tumorigenesis [26]. HDAC inhibitors (HDACis) constitute a new group of epigenetic agents that has gained much attention in cancer drug development in recent years. HDACis exhibit their anticancer activities by inducing cell cycle arrest, cell differentiation, and apoptosis [27]. HDACi treatment increases protein acetylation leading to transcriptional activation of genes involved in cell apoptosis. These inhibitors can up-regulate the expression of both death receptors and their ligands *in vitro *and *in vivo *in transformed cells, but not in normal cells [28]. More than 10 structurally different HDACis are currently (or have been tested) in anti-cancer clinical trials, such as suberoylanilide hydroxamic acid (SAHA), valproic acid (VPA), and PXD-101 [25]. However, resistant to the HDACi treatment has been reported in certain cancer cells in pre-clinical experiments and patients in clinical trials. It is necessary and important to understand the mechanisms of HDACi resistance and develop methods to overcome the resistance [29].

In this study, we demonstrated that TSA, a well-known HDACi, induced ATX expression in various cancer cell lines. HDAC3 and HADC7, as the targets of TSA, were involved in ATX expression regulation in cancer cells. The TSA-induced ATX protected cancer cells from TSA-induced apoptosis by producing LPA through its lysoPLD activity, while BrP-LPA and S32826, the inhibitors of ATX-LPA axis, promoted the TSA-induced apoptosis. These results suggest that inhibition of ATX-LPA axis would potentially improve the efficacy of HDAC inhibitors in cancer treatment.

## Results

### HDAC inhibitors induce ATX expression in cancer cells

**Figure 1:**
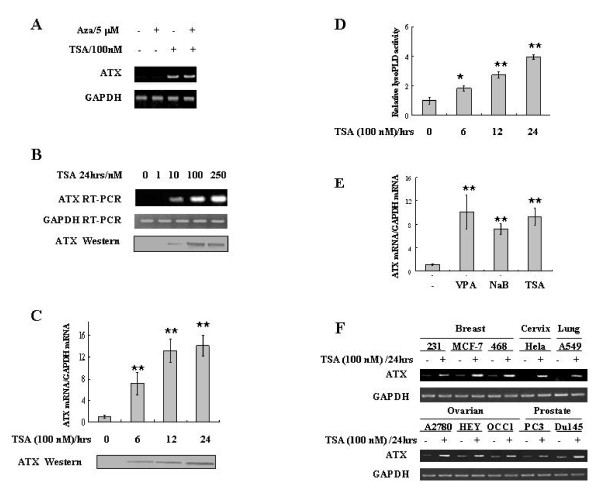
**TSA induces ATX expression in various cancer cell types**. A, SW480 cells were treated with Aza (5 μM) for 48 hrs or TSA (100 nM) for 24 hrs, followed by ATX mRNA detection by RT-PCR. B, SW480 cells were treated with the indicated concentrations of TSA for 24 hrs. The ATX mRNA expression in cells and ATX protein in the conditional medium were detected by RT-PCR and Western blotting analyses, respectively. C, SW480 cells were treated with TSA (100 nM) for 0, 6, 12, or 24 hrs. After TSA treatment, the ATX mRNA expression in cells and secreted ATX protein in the conditional medium were examined by real-time RT-PCR and Western blotting analyses, respectively. D, LysoPLD activity in the conditional medium was determined with FS-3 as substrate after TSA treatment for indicated times. E, SW480 cells were treated with VPA (1 mM), NaB (1 mM), or TSA (100 nM) for 24 hrs. The ATX mRNA expression was evaluated by real-time RT-PCR. F. The indicated breast, cervical, lung, ovarian, and prostate cancer cell lines were treated with or without TSA (100 nM for 24 hrs), and then ATX mRNA expression levels were detected by RT-PCR. The p values derived from Student's t test are (*) p < 0.005, (**) p < 0.001.

To investigate whether ATX expression is epigenetically regulated in cancer cells, we tested the roles of DNA methylation and protein acetylation in ATX expression in a colon cancer cell line SW480, where endogenous ATX expression was not detectable. HDAC inhibitor trichostatin A (TSA), but not 5-aza-2'-deoxycytidine, an inhibitor of DNA methylation, induced ATX expression in SW480 cells (Figure 1A). This induction was dose- and time-dependent at both RNA and protein levels (Figure 1B and Figure 1C). Moreover, TSA induced a time-dependent increase of lysoPLD activity in cell culture medium (Figure 1D), which was correlated well with the increase of secreted ATX protein (Figure 1C). To determine whether the ATX induction ability is limited to TSA, other HDAC inhibitors were used to treat SW480 cells. Similar to TSA, sodium butyrate (NaB) and valproic acid (VPA) also induced ATX expression in SW480 cells (Figure 1E), suggesting that HDAC(s) is involved in ATX expression regulation.

A panel of different cancer cell lines was used to test whether ATX induction by TSA is limited to SW480 cells. TSA induced ATX expression in various cancer cell types, including breast, cervical, lung, ovarian, and prostate cancer cells (Figure 1F). Furthermore, NaB and VPA also could induce ATX expression in various cancer cells other than SW480 (additional file 1). The broad range of ATX induction by HDACi in cancer cells indicates the generality and importance of ATX expression regulation by protein acetylation. However, this effect was not universal, since TSA failed to induce ATX expression in a few cell lines tested, including HT-29, LNcap and Jurkat cells (data not shown).

### Down-regulation of HDAC3 and HDAC7 induces ATX expression in cancer cells

**Figure 2:**
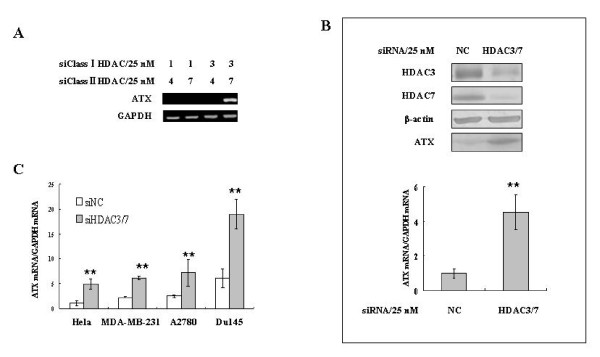
**Knockdown of HDAC3 and HDAC7 up-regulates ATX expression in cancer cells**. A, SW480 cells were co-transfected with the indicated Class I and Class II HDAC siRNAs. ATX mRNA expression was detected by RT-PCR 48 hrs post transfection. B, SW480 cells were co-transfected with HDAC3 and HDAC7 siRNAs. ATX expression at the RNA (in cells) and protein levels (in culture medium) were detected 48 hrs post transfection by real-time RT-PCR and Western blotting, respectively. C, HDAC3 and HDAC7 siRNAs were co-transfected into Hela, MDA-MB-231, A2780, and Du145 cells, respectively, with non-specific siRNA (siNC) as a control. Real-time RT-PCR was used for detecting ATX mRNA expression. The p value derived from Student's t test is (**) p < 0.001.

TSA inhibits the Class I and II, but not Class III HDACs. More specifically, TSA strongly inhibits HDAC1, HDAC2, HDAC3, HDAC4, HDAC6 and HDAC7 [24]. To identify the TSA target(s) involved in ATX induction, HDAC1, HDAC2, HDAC3, HDAC4, HDAC6, and HDAC7 were individually down-regulated with their specific siRNAs. At least 50% down-regulation was achieved for each of these HDACs. However, none of the respective HDAC knockdown led to a significant induction of ATX expression in SW480 cells (additional file 2). These data suggest that either these HDACs are not functionally involved in ATX expression regulation or a combination of more than one HDAC is involved. We tested different combinations of siRNAs against HDACs, and found that only the combination of siRNAs against HDAC3 and HDAC7 induced ATX expression in SW480 cells (Figure 2A). Other Class I and II HDAC siRNA combinations including the siRNAs against HADCs 1 and 4, 1 and 7, or 3 and 4 were ineffective (Figure 2A). The down-regulation of HDAC3 and HDAC7 by co-transfection of their siRNAs was confirmed by Western blot analyses, and ATX expression was tested at the protein level in the conditional medium and the RNA level in SW480 cells (Figure 2B). To verify the effect of HDAC3/HDAC7 on ATX expression further, siRNAs against these two HDACs were co-transfected into several additional cell lines, including Hela, MDA-MB-231, A2780, and Du145 cells. Up-regulation of ATX expression was observed in all of these cell lines when HDAC3 and HDAC7 were down-regulated together (Figure 2C). Collectively, our data indicate that HDAC3 and HDAC7 play a critical negative regulatory role of in ATX expression, and serve as TSA targets in the ATX induction in cancer cells.

### Exogenous over-expression of HDAC7 inhibits ATX expression in cancer cells in a HDAC3-dependent manner

**Figure 3:**
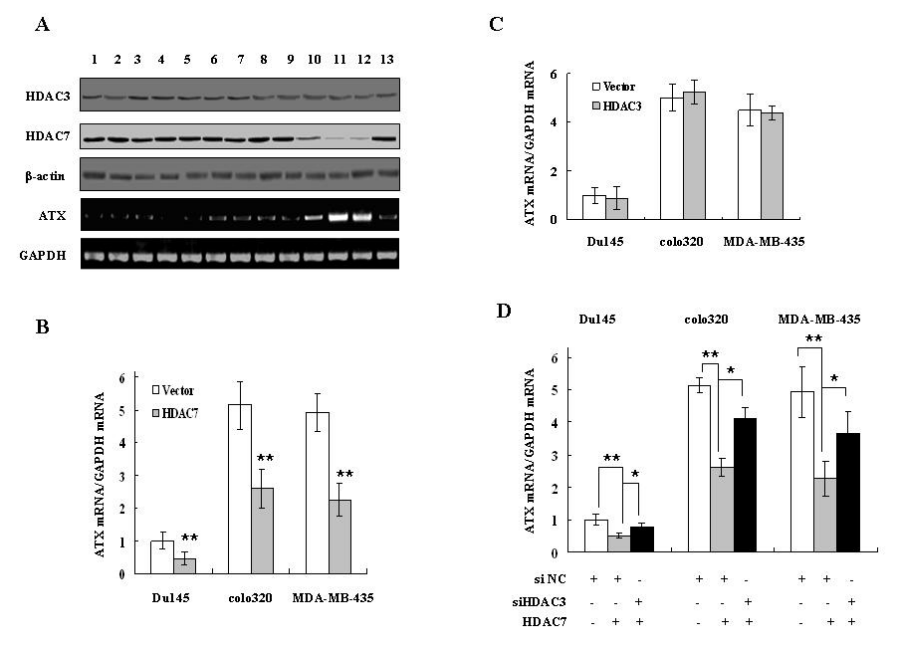
**Exogenous over-expression of HDAC7 inhibits ATX expression in cancer cells in a HDAC3-dependent manner**. A, HDAC3 and HDAC7 protein levels, as well as ATX mRNA levels, were detected by Western blot and RT-PCR analyses, respectively, in 13 cancer cell lines (1, MDA-MB-231; 2, MCF-7; 3, MDA-MB-468; 4, Hela; 5, A549; 6, A2780; 7, HEY; 8, OCC1; 9, PC3; 10, Du145; 11, MDA-MB-435; 12, colo320; 13, HT-29). B and C, HDAC7 (B) or HDAC3 (C) over-expression plasmid was transfected into indicated cancer cells with empty vector as the control. ATX mRNA expression was detected by real-time RT-PCR 48 hrs post transfection. D, The indicated cancer cells were first transfected with non-specific siRNA (siNC) or HDAC3 siRNA for 24 hrs, followed by transfection of the HDAC7 over-expression plasmid. ATX mRNA expression levels were detected by real-time RT-PCR 48 hrs post the second transfection. The p values derived from Student's t test are (*) p < 0.005, (**) p < 0.001.

To expand our studies on the role of HDACs in ATX expression, we examined expression levels of HDAC3, HDAC7 and ATX in 13 cancer cell lines. The levels of HDAC3 expression were almost similar in all of cell lines tested, while the levels of HDAC7 expression had an inverse correlation with those of ATX expression. In particular, in 10 cancer cells with relatively high HDAC7 expression, the endogenous ATX expression is low or undetectable (Figure 3A, lines 1-9 and line 13).

The functional effects of HDAC7 and HDAC3 on ATX expression were examined further using a gain-of-function approach in Du145, colo320, and MDA-MB-435 cells, where low levels of endogenous HDAC7, but high levels of endogenous ATX, were expressed (Figure 3A, lines 10 to 12). Exogenous over-expression of HDAC7 significantly inhibited ATX expression in these cancer cells (Figure 3B), suggesting that HDAC7 is an important negative regulator of ATX expression. On the other hand, exogenous over-expression of HDAC3 did not affect ATX expression (Figure 3C), but the functional involvement of HDAC3 in ATX regulation was confirmed by its down-regulation. When the Du145, colo320 or MDA-MB-435 cells were pre-transfected with HDAC3 siRNA, the ATX expression inhibited by HADC7 over-expression was significantly restored (Figure 3D). These data indicate that the exogenous over-expression of HDAC7 inhibits ATX expression in a HDAC3-dependent manner, and suggest that HDAC7 and HDAC3 collaboratively suppress ATX expression in cancer cells.

### TSA-induced ATX protects cancer cells against TSA-induced apoptosis through its lysoPLD activity

**Figure 4:**
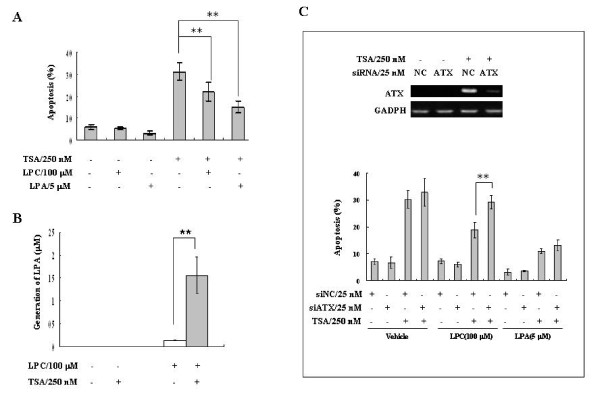
**The TSA-induced ATX protects cancer cells from TSA-induced apoptosis through its lysoPLD activity**. A, SW480 cells were treated with or without TSA (250 nM) for 24 hrs in the presence of LPC (100 μM) or LPA (5 μM) in the serum-free conditional medium containing 250 μg/ml fatty-acid free BSA, and then the apoptosis of SW480 cells were measured. B, SW480 cells were treated with or without TSA (250 nM) for 24 hrs, and then the concentrated conditional media (30-fold) were incubated with 100 μM LPC (18:1) for 6 hrs at 37°C. Lipids were extracted and analyzed by liquid chromatography-mass spectrometry (LC-MS). The levels of LPA (18:1) were obtained from three experiments. C, ATX siRNA was transfected into SW480 cells to block the ATX induction by TSA with non-specific siRNA (siNC) as the control. After siRNA transfection for 48 hrs, SW480 cells were treated with or without TSA (250 nM) in the presence of LPC (100 μM) or LPA (5 μM). The apoptosis of SW480 cells was measured after TSA treatment for 24 hrs. The p value derived from Student's t test is (**) p < 0.001.

SW480 cells are HDACi sensitive [30] with ~30% cells undergoing apoptosis after TSA (250 nM) treatment for 24 hrs in the serum-free conditional medium. Since the ATX-LPA axis has been shown to be involved in cell survival and/or proliferation in many cancer cell types [3], we tested whether LPC (the substrate of ATX) or LPA (the product of ATX) had effects on TSA-induced cell apoptosis. In the presence of LPC (100 μM) or LPA (5 μM), SW480 cells were protected against the TSA-induced apoptosis (Figure 4A). As described above, the secreted ATX protein and the lysoPLD activity in culture medium were increased upon TSA treatment (Figure 1C and Figure 1D). We checked the LPA production by liquid chromatography/Mass spectrometry (LC-MS) analysis after incubating the conditional culture medium with ATX substrate LPC (100 μM, 18:1-LPC). In the presence of LPC, the medium of SW480 cells without TSA treatment contained low levels of LPA (0.13 ± 0.01 μM, 18:1-LPA), while the medium of TSA-treated SW480 cells increased the LPA levels by about 12.0-fold (1.56 ± 0.40 μM 18:1-LPA, P < 0.001) (Figure 4B). These data suggest that LPC is converted to LPA by TSA-induced ATX to protect cells from apoptosis. Indeed, when ATX was down-regulated by siRNA in SW480 cells to block the TSA-induced ATX expression, the protective effect of LPC, but not that of LPA, against TSA-induced cell apoptosis was significantly reduced (Figure 4C). Therefore, HDACis, including TSA, have dual effects to induce cell apoptosis and ATX expression at the same time, and ATX can antagonize the HDACi-induced apoptosis by producing LPA through its lysoPLD activity.

### ATX-LPA axis protects cells from TSA-induced apoptosis via cell-specific LPA receptors

**Figure 5:**
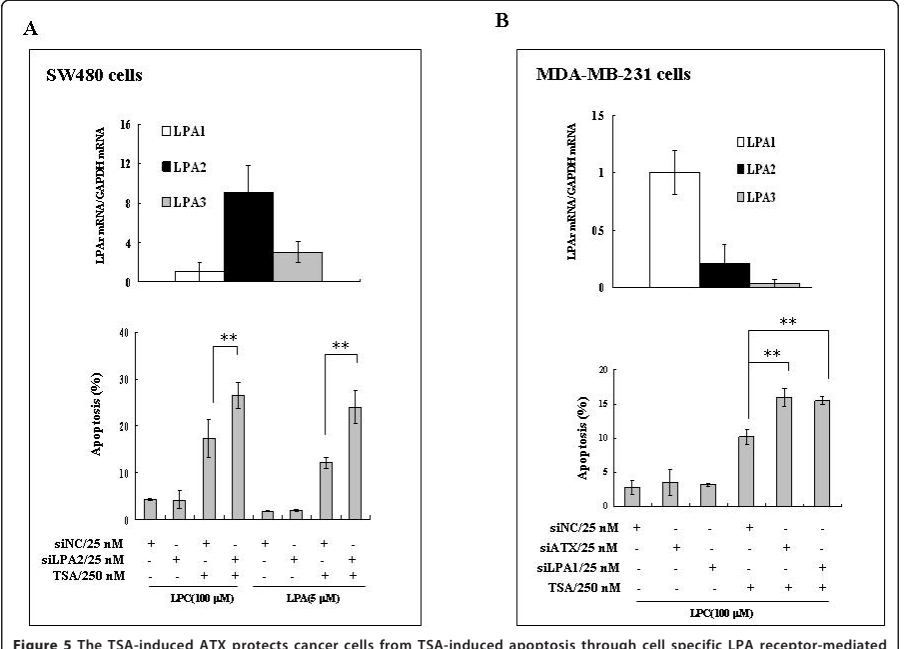
**The TSA-induced ATX protects cancer cells from TSA-induced apoptosis through cell specific LPA receptor-mediated signaling**. A, The mRNA expression levels of LPA_1_, LPA_2 _and LPA_3 _in SW480 cells were examined by real-time RT-PCR. SW480 cells were transfected by LPA_2 _siRNA with non-specific siRNA (siNC) as control. After siRNA transfection for 48 hrs, SW480 cells were treated with or without TSA (250 nM) in the presence of LPC (100 μM) or LPA (5 μM). The apoptosis of SW480 cells were measured after TSA treatment for 24 hrs. B, The mRNA expression levels of LPA_1_, LPA_2 _and LPA_3 _in MDA-MB-231 cells were examined by real-time RT-PCR. MDA-MB-231 cells were transfected by ATX or LPA_1 _siRNA with non-specific siRNA (siNC) as control. After siRNA transfection for 48 hrs, MDA-MB-231 cells were treated with or without TSA (250 nM) in the presence of LPC (100 μM). The apoptosis of MDA-MB-231 cells was measured after TSA treatment for 24 hrs. The p value derived from Student's t test is (**) p < 0.001.

The biological function of ATX is to convert LPC into LPA, a bioactive lysophospholipid that acts on specific G protein-coupled receptors (GPCRs) to perform various biological activities, including the anti-apoptosis activity. Quantitative PCR analyses showed that LPA_2 _was the dominant LPA receptor expressed in SW480 cells (Figure 5A). Down-regulation of LPA_2 _by siRNA enhanced TSA-induced cell apoptosis in the presence of LPC or LPA (Figure 5A), while down-regulation of either LPA_1 _or LPA_3 _did not have such an effect (data not shown), indicating that LPA_2 _is involved in the protective effect of ATX-LPA signaling.

Similar to SW480 cells, MDA-MB-231 cells were sensitive to TSA and were protected by LPA and LPC from the TSA-induced apoptosis (data not shown). In MDA-MB-231 cells, LPA_1 _was the dominant LPA receptor expressed (Figure 5B). The protective effect of LPC against TSA-induced MDA-MB-231 cell apoptosis was significantly inhibited by the down-regulation of ATX or LPA_1 _(Figure 5B), but not by that of LPA_2 _or LPA_3 _(data not shown), indicating that ATX-LPA axis protects MDA-MB-231 cell from the TSA-induced apoptosis through the LPA_1_-mediated signaling. Collectively, our data suggest that the cell-specific LPA receptor(s) is involved in the LPC-ATX-LPA signaling axis, which plays a role in reducing the efficacy of apoptosis induction by TSA and potentially other HDACi.

### Inhibitor of the ATX-LPA axis enhances the TSA-induced cell apoptosis

**Figure 6:**
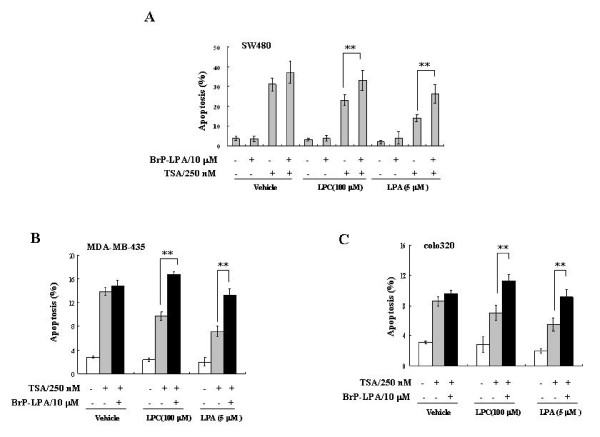
**Inhibitor of ATX-LPA signaling enhances the TSA-induced cell apoptosis**. A, The SW480 cells were pretreated with BrP-LPA (10 μM) for 1 hr, and then treated with TSA (250 nM) in the presence of LPC (100 μM) or LPA (5 μM). The apoptosis of SW480 cells were measured after TSA treatment for 24 hrs. B and C, MDA-MB-435 (B) and colo320 cells (C) were treated with TSA (250 nM) with or without the pre-treatment of BrP-LPA (10 μM) in the presence of indicated lipids in serum-free conditional medium. The cell apoptosis was measured after TSA treatment for 24 hrs. The p value derived from Student's t test is (**) p < 0.001.

HDACis have been rapidly moved from the laboratory bench to clinical trials as novel anticancer agents [27]. As described above, the TSA-induced ATX could convert LPC to LPA through its lysoPLD activity and protect the cancer cells from TSA-induced apoptosis via LPA receptor-mediated signaling. Furthermore, we test whether ATX-LPA axis inhibition would increase the efficacy of apoptosis induction by TSA. BrP-LPA is a potent ATX inhibitor and a pan LPA receptor antagonist, which not only inhibits the ATX lysoPLD activity but also blocks LPA receptor-mediated signaling [31-33]. As predicted, the lysoPLD activity in the conditional culture medium of TSA-treated SW480 cells was significantly inhibited by BrP-LPA (additional file 3A). In the presence of either LPC or LPA, combination of TSA and BrP-LPA induced higher percentage of apoptotic death in SW480 cells, compared with treatment with TSA alone (Figure 6A). In the presence of either LPC or LPA, MDA-MB-435 and colo320 cells, which express high endogenous levels of ATX, were relatively resistant to TSA treatment, while addition of BrP-LPA significantly enhanced the TSA-induced apoptosis in MDA-MB-435 and colo320 cells (Figure 6B and Figure 6C). S32826, another ATX inhibitor with the IC_50 _in nanomolar range (additional file 3B) [34], was used to testify the effects of combinational treatment with ATX inhibitor and TSA. In the presence of LPC, S32826 obviously enhanced the TSA-induced apoptosis in SW480, colo320, MDA-MB-231 and MDA-MB-435 cells (additional file 4). However, compared with BrP-LPA, S32826 could not promote cell apoptosis in the presence of LPA because S32826 is not a LPA receptor antagonist (additional file 4A). These data suggest that the ATX-LPA axis inhibition is able to improve the efficacy of cancer treatment with HDACi.

## Discussion

ATX is the primary enzyme to produce LPA, a bioactive phospholipid inducing cell proliferation, survival and migration. ATX-LPA signaling axis is critically involved in the development and progression of several cancers. It is found that ATX is highly expressed in some cancer cells and contributes to their tumorigenesis, invasion, and metastases, while in other cancer cells ATX is silenced or expressed at low level [23]. In the present paper, we have demonstrated that HDAC3 and HDAC7 are involved in the endogenous ATX expression regulation, which is supported by the loss- and gain-of-function studies in multiple cancer cell lines. Down-regulation of both HDAC3 and HDAC7 up-regulated ATX expression in the cancer cells with low ATX expression, while over-expression of HDAC7 inhibited ATX expression in a HDAC3-dependent manner in the cancer cells with high endogenous ATX expression, indicating that HDAC3 and HDAC7 collaboratively suppress ATX expression in cancer cells. There is evidence that the HDAC activity of HDAC7 is dependent on the interaction with HDAC3, which is mediated by the transcriptional corepressor SMRT/N-CoR [35]. Therefore, it is possible that the HDAC3/HDAC7 complex is involved in ATX expression regulation.

In the 13 tested cancer cell lines, HDAC3 was almost constitutively expressed, but the expression levels of HDAC7 were inversely correlated to those of ATX, suggesting that expression regulation of HDAC7 may play an important role in the differential expression of ATX observed in different cancer cell lines. It has been reported that ATX expression is inducible by several growth factors, such as VEGF [13,36] and BMP-2 [37]. Both VEGF and BMP-2 can enhance the phosphorylation of HDAC7 via protein kinase D, which promotes nuclear export of HDAC7 [38,39]. Thus, it will be interesting to test whether HDAC7 plays a role in the ATX expression regulation by VEGF and BMP-2.

Acetylated histones are the major targets of HDACs, and HDACi treatment leads to increased histone acetylation. Inhibiting HDACs by TSA leads to increased histone acetylation, and histone H3 lysine 9 acetylation is a hallmark of the TSA-induced histone acetylation [40]. We tested whether increased histone acetylation was sufficient to induce ATX expression. In HT-29 cells, TSA induced histone H3 acetylation, but not ATX expression (additional file 5A). In SW480 cells, down-regulation of HDAC3 alone, but not HDAC7, led to histone H3 acetylation in the ATX promoter, while down-regulation of both of these two HDACs was necessary to induce ATX expression (additional file 5B). These data suggest that histone acetylation in the ATX promoter is insufficient to up-regulate ATX expression. A number of non-histone proteins have been identified as substrates of HDACs, among which there are transcription factors and/or transcription regulators involved in gene expression regulation [25]. The target protein(s) of HDAC3/HDAC7 involved in ATX expression regulation remains to be identified, which will be helpful to explain why TSA cannot induce ATX expression in a few cancer cell lines including HT-29, LNcap and Jurkat cells.

HDACs play an important role in the cancer pathogenesis, and HDAC-dependent aberrant transcriptional repression is implicated as one of the main oncogenic mechanisms. As a result, HDAC inhibitors have been evaluated in clinical trials for solid tumors and hematological malignancies [41]. However, resistant to the HDACi becomes a major obstacle of these reagents. The proposed mechanisms of HDACi resistance include up-regulation of cellular antioxidant pathways, increased expression of the anti-apoptotic protein Bcl-2 and the stress-responsive transcription factor NF-κB, and the alternative gene silencing pathways such as DNA methylation [42]. The current study illustrates that ATX induction by HDACi is a novel mechanism with a strong internal antagonism against the apoptotic effects of HDACi on cancer cells. As a potential HDACi, TSA leads to apoptotic cell death by inhibiting HDACs, but releases the repression of ATX expression at the same time by targeting HDAC3 and HDAC7. The induced ATX produces LPA from LPC through its lysoPLD activity and antagonizes the TSA-induced cell apoptosis through the signaling pathway mediated by particular cell-specific LPA receptor. As a rich source of both LPC (the substrate of ATX) and LPA (the product of ATX), serum protected MDA-MB-231 cells from TSA-induced apoptosis significantly. In the serum-containing medium, both ATX inhibitor S32826 and LPA_1/3 _inhibitor Ki16425 could enhance the TSA-induced apoptosis of MDA-MB-231 cells, in which LPA_1 _is involved in the protective effect of ATX-LPA signaling (additional file 6). These data implicate that, besides the anti-apoptotic cytokines and growth factors in serum, the LPC-ATX-LPA axis plays a role to protect cancer cells from TSA-induced apoptosis.

ATX is secreted through the classical secretory pathway [2], and is rapidly taken up from circulation and degraded by a general mechanism of the liver LSEC scavenger system [5], which leaves little space for specific interference with its secretion or promotion of its clearance. Therefore, the exploring of ATX inhibitors is highly focused in cancer therapy, and several ATX specific inhibitors have been developed in recent years [43-45]. In the present paper, we have demonstrated that inhibition of ATX lysoPLD activity with ATX inhibitor (such as BrP-LPA and S32826) could enhance the TSA-induced apoptosis in cancer cells. The combinational treatment with ATX inhibitor and HDACi would be helpful to improve the efficacy of HDACi-based therapeutics against cancer.

## Conclusions

In the present paper, we demonstrated a novel ATX expression regulation mechanism in cancer cells, in which HDAC3 and HDAC7 were involved as negative regulators. During TSA treatment, ATX was induced due to the HDAC3 and HDAC7 inhibition and functionally antagonized the TSA-induced apoptosis. The use of TSA combining with an ATX-LPA axis inhibitor resulted in the increased apoptotic response in cancer cells. These findings are significant to reveal an internal HDACi-resistant mechanism generally existing in cancer cells, and helpful to develop new combinational approach to improve the efficacy of HDACi as chemotherapeutic agent in cancer treatment.

## Methods

### Materials and reagents

5-AZA-2'-deoxycytidine, trichostatin A (TSA), sodium butyrate (NaB), and Valproic acid (VPA) were purchased from Sigma-Aldrich (Saint Louis, MI, USA). FS-3 (lysoPLD/Autotaxin substrate), 1-Bromo-3(S)-hydroxy-4-(palmitoyloxy) butylphosphonate (BrP-LPA) and 4-(Tetradecanoylamino) benzyl phosphonic acid disodium salt (S32826, ATX inhibitor) were obtained from Echelon Biosciences (Salt Lake City, Utah). LPA_1/3 _inhibitor Ki16425 was obtained from Cayman Chemical (Ann Arbor, MI, USA). 18:1 lysophosphatidylcholine (LPC) and 18:1 lysophosphatidic acid (LPA) were from Avanti Polar Lipid Inc. (Alabaster, AL). The HDAC7 expression plasmid was a kind gift from Dr. Hung-Ying Kao [46], and the HDAC3 expression plasmid was from Dr. Edward Seto's lab [47].

### Antibodies

The ATX primary antibody was generated as described previously [48]. The primary antibodies against HDAC7 and β-actin were purchased from Cell Signaling Technology (Beverly, MA). The primary antibodies against HDAC3 and HRP conjugated secondary antibody were from Santa Cruz Biotechnology (Santa Cruz, CA). The antibodies against total histone H3 and histone H3 Lys9 acetylation were obtained from Millipore (Billerica, MA).

### Cell Cultures, siRNA and transfections

The SW480, MDA-MB-231, MCF-7, MDA-MB-468, Hela, A549, MDA-MB-435 and HT-29 cells were cultured in Dulbecco's modified Eagle's medium (Hyclone). A2780, HEY, OCC1, PC-3, Du145, and colo320 cells were maintained in RPMI-1640 medium (Hyclone). Mediums were supplemented with 10% fetal bovine serum (Hyclone), 2 mM L-glutamine (Gibco), 100 μg/ml streptomycin (Gibco) and 100 U/ml penicillin (Gibco). All cells were cultured at 37°C in a humidified atmosphere containing 5% CO_2_. For experiments to detect the effects of LPA and LPC on cell apoptosis, the cancer cells were cultured in a conditional serum-free medium with 250 μg/ml fatty-acid free BSA as described previously [48]. All siRNAs were synthesized in GenePharma (Shanghai, China), and the target sequences were: ATX-GUGGACCAAUCUUCGACUA; LPA_1_-GAAAUGAGCGCCACCUUUA; LPA_2_-GGUCAAUGCUGCUGUGUAC; LPA_3_-CAGCAGGAGUUACCUUGUU; HDAC1-GCUUCAAUCUAACUAUCAA; HDAC2-CAGUGAUGAGUAUAUCAAA; HDAC3-GCCGGUUAUCAACCAGGUA; HDAC4-CGUCAACAUGGCUUUCACC; HDAC6-GCUCGGCCAAGCAAUGGAA; HDAC7-UCACUGACCUCGCCUUCAA; non-specific-UUCUCCGAACGUGUCACGU. The siRNAs were transfected into cells with lipofectamine 2000 (Invitrogen), according to the protocol supplied by manufacturer. In each siRNA transfection experiment, the non-specific siRNA was used as control.

### RNA Extraction, RT-PCR and Real-time RT-PCR

Total RNA was extracted from cancer cells with Trizol (Invitrogen), and then digested with DNase I (Ambion) for 15 min at 37°C in order to remove DNA contamination. RNA (2 μg) from cancer cells were reverse-transcribed using anchored oligo dT (15) primers and the Reverse Transcription System (Promega). The cDNAs encoding indicated genes were amplified with specific primers. ATX primers: 5'-TATGCTTCGGAAAGAAATGGAG-3' and 5'-ATGTTCAATGTCACGCACCCT-3'; GAPDH primers: 5'-TTAGC ACCCCTGTCCAAGG-3' and 5'-CCTACTCCTTGGAGGCCATG-3'; HDAC1 primers: 5'-GAACTGGGGACCTACGGG-3' and 5'-GCTCTTGACAAATTCCACACAC-3'; HDAC2 primers:5'-AGTTGCCCTTGATTGTGAGA-3' and 5'-CCACTGTTGTCCTTGGATTTAT-3'; HDAC3 primers:5'-TGATGACCAGAGTTACAAGCAC-3' and 5'-GGGCAACATTTC GGACAG-3'; HDAC4 primers:5'-GTGCTGGTGTCATCAGGCTT-3' and 5'-AAATGGCG GTCAGGTCGT-3'; HDAC6 primers:5'-TGCTGTGACACCACTGCCC-3' and 5'-TTCT GGTGGGCGATGTTCTT-3'; HDAC7 primers 5'-GGATTTGATGCTGCTGAGGG-3' and 5'-CCACAGAGAGGGACGCCAG-3'; LPA_1 _primers:5'-CGGCGGGTAGTGGTGGTC-3' and 5'-GTCGCGGTAGGAGTAAATGATG-3'; LPA_2 _primers: 5'-GTCGAGCCTGCTTG TCTTCC-3' and 5'-CCAGGAGCAGTACCACCTG-3'; LPA_3 _primers: 5'-GACGGTGA TGACTGTCTTAGGGG-3' and 5'-GAGGACTGTGGAGGGGATGC-3'. The PCR conditions were: 20 s at 95°C, 20 s for annealing, 25 s at 72°C for reasonable cycles, with a final extension for 5 min at 72°C. Each RT-PCR experiment was repeated at least three times with 3 parallel samples. The real-time RT-PCR was performed using the iQ SYBR Green Supermix (Bio-Rad) with the iCycler iQ real-time RT-PCR detection system (Bio-Rad). Relative expression of each target gene was estimated by normalization with the expression of GAPDH.

### ATX lysoPLD activity analyses

The conditional serum-free medium from cancer cells with or without exposure to TSA was concentrated (30-fold) using Amicon Ultra 30,000 (Millipore). The lysoPLD activity in the concentrated conditional medium was analyzed using fluorogenic substrate FS-3 as described previously [48]. Briefly, the assays were performed by mixing 50 μl concentrated medium with 10 μM FS-3 at 37°C for 4 hrs. LysoPLD activity was measured by detecting the fluorescence increase with 494 and 520 nm as the excitation and emission wavelengths, respectively.

### Lipid extraction and analyses

Lipids were extracted from concentrated (30-fold) conditional medium of SW480 cells with or without TSA treatment, and analyzed with liquid chromatography-tandem mass spectrometry (LC-MS/MS) as described previously [22].

### Western blotting

Cells were lysed in RIPA buffer for 30 min. After centrifugation, the supernatants were quantified by bicinchoninic acid assay (Micro BCA; Pierce Biotechnology, Rockford, IL). For experiments detecting the secreted ATX protein, the culture medium was concentrated (by approximately 30-fold) using Amicon Ultra 30,000 (Millipore). Protein quantification was conducted and equal amount protein was loaded for each sample. Protein samples were subjected to SDS-PAGE and analyzed as described previously [48]. Each Western blot analysis was repeated at least three times.

### Chromatin immunoprecipitation (ChIP)

ChIP assay was performed with a commercial Kit (Millipore 17-371). Briefly, formaldehyde was added to the medium to a final concentration of 1% and incubated for 10 min at room temperature, followed by addition of glycine (0.125 M) and incubation for another 5 min. The fixed cells were scraped into conical tubes, pelleted, and lysed in a SDS lysis buffer containing protease inhibitor (Roche). DNA was sheared to fragments of 500-1000 bp by sonication. The chromatin was precleared with salmon sperm DNA/protein G-agarose slurry for 90 min at 4°C. The precleared supernatant was incubated with antibodies against acetylation histone H3 (K9), histone H3 or normal rabbit IgG overnight at 4°C. The immunocomplexes were eluted with elution buffer (1% SDS and 0.1 M NaHCO_3_) after washing. NaCl was added into eluted samples (final concentration 0.2 M) to reverse histone-DNA cross-links and the samples were heated for 4 hrs at 65°C. The purified DNA was used for PCR reactions. Primers for PCR amplification of the ATX promoter DNA were 5'-ATGATAGCTTAAGCCTCTTAGG-3' and 5'-TGCAGCGTGTTCTCTTTGCCTT-3'. PCR was carried out for 35 cycles (95°C for 40 s, 60°C for 40 s, and 72°C for 40 s), and PCR products were resolved on 2% agarose gel.

### Measurement of apoptosis

Cells were subjected to TSA treatment in the serum-free conditional medium containing 250 μg/ml fatty-acid free BSA. To test the effects of LPC and LPA on TSA-induced apoptosis, cells were starved for 16 hrs in serum-free conditional medium before the TSA treatment in the presence of LPA or LPC. Apoptosis assays of the TSA-treated cells were performed by PI staining and FACS analysis as described previously [30,49].

### Statistical analysis

Data was analyzed by Student's t-test for two-group comparison. The values shown in the graph are the mean ± S.D, and the p value derived from Student's t test is (*) p < 0.005, (**) p < 0.001.

## Abbreviations

ATX: Autotaxin; TSA: trichostatin A; HDAC: Histone deacetylase; HDACi: HDAC inhibitor; LysoPLD: lysophospholipase D; BrP: LPA-butylphosphonate

## Competing interests

The authors declare that they have no competing interests.

## Authors' contributions

Conceived and designed the experiments: JZ. Performed the experiments: SL and BW; Data analyzed: SL, YX and JZ. Paper writing: SL, YX and JZ. All authors have read and approved the final manuscript.

## Supplementary Material

Additional file 1 **figure S1 - HDAC inhibitors, NaB and VPA, upregulate ATX expression in different cancer cells**. Hela, MDA-MB-231 and Du145 cells were treated with NaB (1 mM) or VPA (1 mM) for 24 hrs, and then the ATX mRNA expression levels were evaluated by RT-PCR. Click here for file

Additional file 2 **figure S2 - Knockdown of individual HDAC alone could not up-regulate ATX expression**. SW480 cells were transfected with the indicated HDAC siRNA with non-specific siRNA (siNC) as control. Total RNA was extracted at 48 hrs post transfection, and then the mRNA expression levels of HDACs and ATX in SW480 cells were detected by RT-PCR. Click here for file

Additional file 3 **figure S3 - BrP-LPA and S32826 inhibited ATX lysoPLD activity**. SW480 cells were treated with or without TSA (100 nM) for 24 hrs. After treatment, the conditional culture medium was collected and concentrated about 30-fold. Different doses of BrP-LPA (A) or S32826 (B) were added to the concentrated conditional medium as indicated. After incubation for 1 hr, LysoPLD activity in the conditional medium was determined with FS-3 as substrate as described in Methods. Click here for file

Additional file 4 **figure S4 - ATX inhibitor S32826 enhanced the TSA-induced cell apoptosis**. The SW480 (A), MDA-MB-231 (B), colo320 (C) and MDA-MB-435 (D) cells were pretreated with or without ATX inhibitor S32826 (1 μM) for 1 hr, and then treated with TSA (250 nM) in the presence of LPC (100 μM) or LPA (5 μM) as indicated. The cell apoptosis was measured after TSA treatment for 24 hrs. The p values derived from Student's t test are (*) p < 0.005, (**) p < 0.001. Click here for file

Additional file 5 **figure S5 - Histone acetylation in ATX promoter region is insufficient to up-regulate ATX expression**. A, SW480 and HT-29 cells were treated with or without TSA (100 nM) for 24 hrs, and then subjected to ChIP assays as described in Methods to detect the acetylated histone H3 in the ATX promoter region. ATX expression levels were detected by RT-PCR. B, SW480 cells were transfected with HDAC3 and/or HDAC7 siRNA(s) as indicated. The ChIP assays were performed 48 hrs post transfection to detect the acetylated histone H3 in ATX promoter region. The acetylation level of histone H3 (Lys9) in SW480 cell lysates was detected by Western blot analyses and normalized by the total histone H3. Click here for file

Additional file 6 **figure S6 - Inhibition of ATX-LPA signaling enhanced TSA-induced apoptosis in serum-containing medium**. The MDA-MB-231 cells were treated with TSA (1 μM) for 48 hrs in the conditional serum-free medium or serum (10%)-containing medium to detect the effect of serum on TSA-induced apoptosis. Furthermore, in serum (10%)-containing medium, MDA-MB-231 cells were pretreated with ATX inhibitor S32826 (1 μM) or LPA_1/3 _inhibitor Ki16425 (1 μM) for 1 hr, and then treated with TSA (1 μM) for 48 hrs. The cell apoptosis was measured after TSA treatment. The p value derived from Student's t test is (**) p < 0.001. Click here for file
