# Supplementary material for: Autotaxin is induced by TSA through HDAC3 and HDAC7 inhibition and antagonizes the TSA-induced cell apoptosis
Source: Mol Cancer. 2011 Feb 12;10:18. doi: 10.1186/1476-4598-10-18 (PMC3055229; doi:10.1186/1476-4598-10-18)

Supplementary figure 1– HDAC inhibitors, NaB and VPA, upregulate ATX expression in different cancer cells.

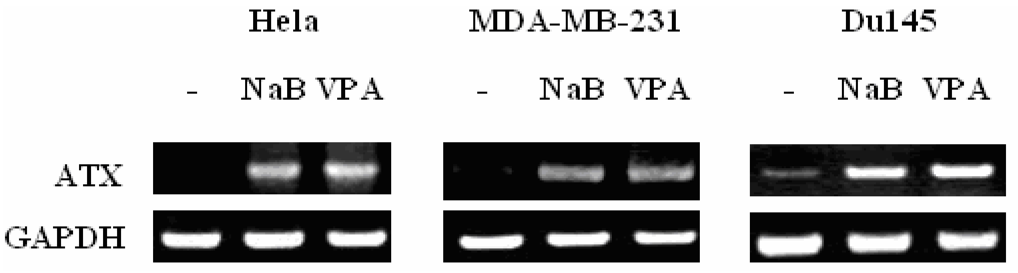

Supplement: Additional file 1 — figure S1 - HDAC inhibitors, NaB and VPA, upregulate ATX expression in different cancer cells. Hela, MDA-MB-231 and Du145 cells were treated with NaB (1 mM) or VPA (1 mM) for 24 hrs, and then the ATX mRNA expression levels were evaluated by RT-PCR. [file 1476-4598-10-18-S1.PDF]
