# Supplementary material for: Autotaxin is induced by TSA through HDAC3 and HDAC7 inhibition and antagonizes the TSA-induced cell apoptosis
Source: Mol Cancer. 2011 Feb 12;10:18. doi: 10.1186/1476-4598-10-18 (PMC3055229; doi:10.1186/1476-4598-10-18)

Supplementary figure 2– Knockdown of individual HDAC alone could not up-regulate ATX expression.

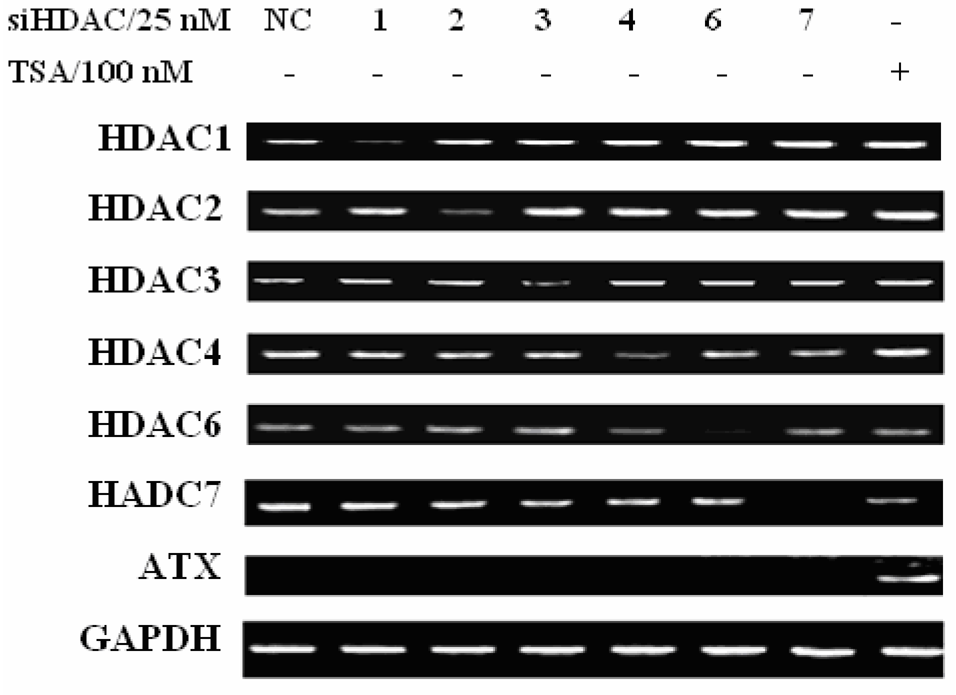

Supplement: Additional file 2 — figure S2 - Knockdown of individual HDAC alone could not up-regulate ATX expression. SW480 cells were transfected with the indicated HDAC siRNA with non-specific siRNA (siNC) as control. Total RNA was extracted at 48 hrs post transfection, and then the mRNA expression levels of HDACs and ATX in SW480 cells were detected by RT-PCR. [file 1476-4598-10-18-S2.PDF]
