# Supplementary figures and images for: Autotaxin is induced by TSA through HDAC3 and HDAC7 inhibition and antagonizes the TSA-induced cell apoptosis
Source: Mol Cancer. 2011 Feb 12;10:18. doi: 10.1186/1476-4598-10-18 (PMC3055229; doi:10.1186/1476-4598-10-18)

Supplementary figure 3— BrP-LPA and S32826 inhibited ATX lysoPLD activity.

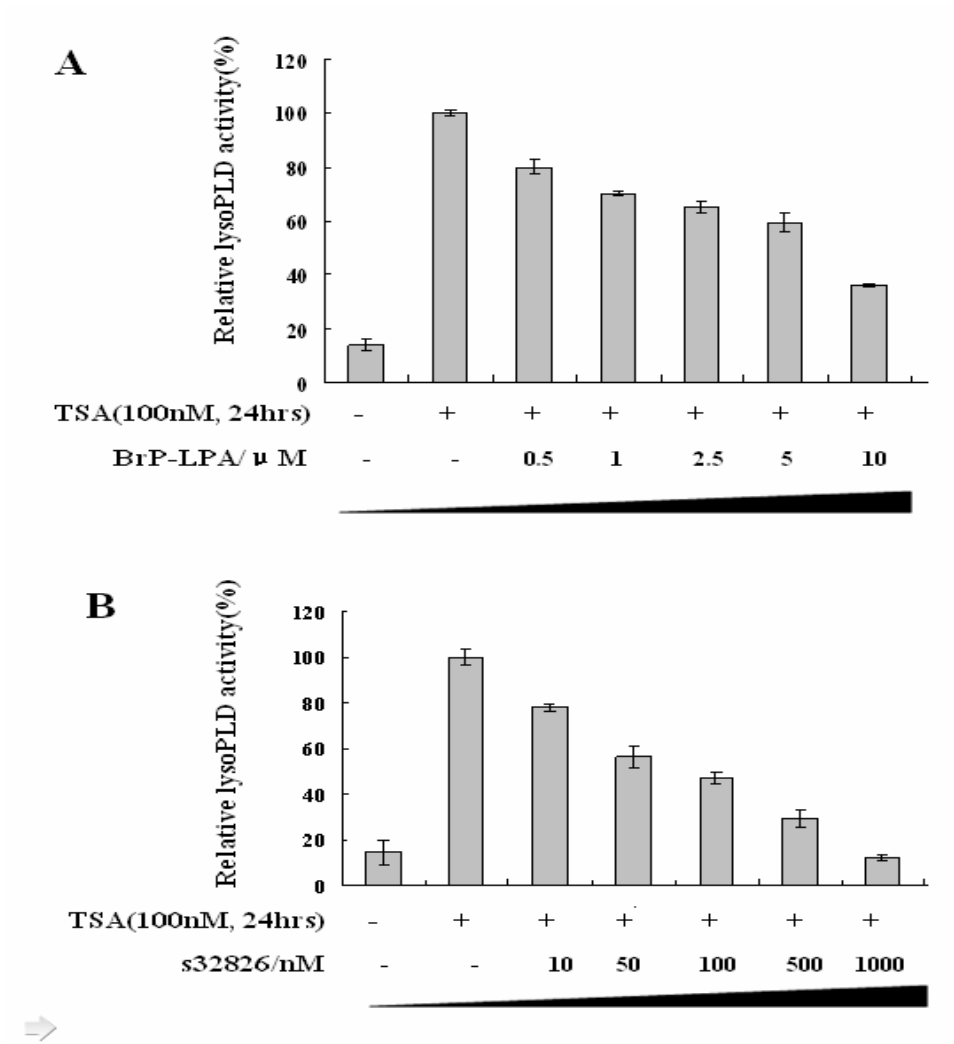

Supplement: Additional file 3 — figure S3 - BrP-LPA and S32826 inhibited ATX lysoPLD activity. SW480 cells were treated with or without TSA (100 nM) for 24 hrs. After treatment, the conditional culture medium was collected and concentrated about 30-fold. Different doses of BrP-LPA (A) or S32826 (B) were added to the concentrated conditional medium as indicated. After incubation for 1 hr, LysoPLD activity in the conditional medium was determined with FS-3 as substrate as described in Methods. [file 1476-4598-10-18-S3.PDF]

Supplementary figure 4– ATX inhibitor S32826 enhanced the TSA-induced cell apoptosis.

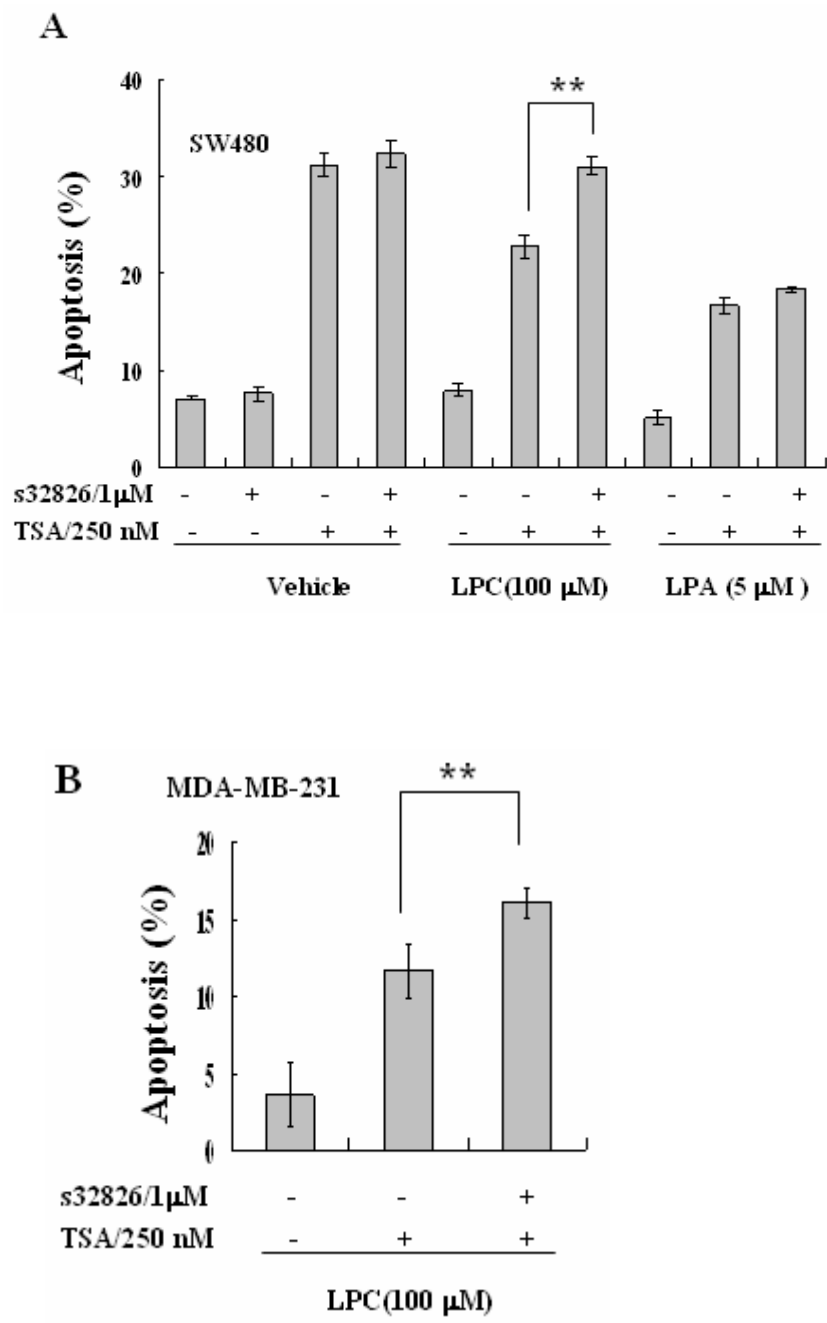

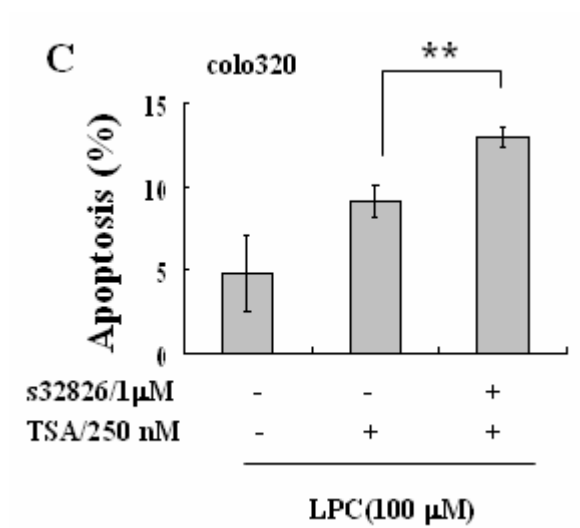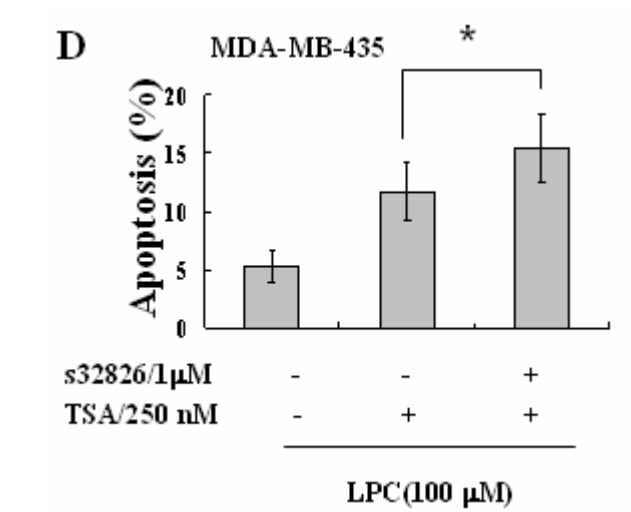

Supplement: Additional file 4 — figure S4 - ATX inhibitor S32826 enhanced the TSA-induced cell apoptosis. The SW480 (A), MDA-MB-231 (B), colo320 (C) and MDA-MB-435 (D) cells were pretreated with or without ATX inhibitor S32826 (1 μM) for 1 hr, and then treated with TSA (250 nM) in the presence of LPC (100 μM) or LPA (5 μM) as indicated. The cell apoptosis was measured after TSA treatment for 24 hrs. The p values derived from Student's t test are (*) p < 0.005, (**) p < 0.001. [file 1476-4598-10-18-S4.PDF]
