# Supplementary material for: Autotaxin is induced by TSA through HDAC3 and HDAC7 inhibition and antagonizes the TSA-induced cell apoptosis
Source: Mol Cancer. 2011 Feb 12;10:18. doi: 10.1186/1476-4598-10-18 (PMC3055229; doi:10.1186/1476-4598-10-18)

Supplementary figure 5– Histone acetylation in ATX promoter region is insufficient to up-regulate ATX expression.

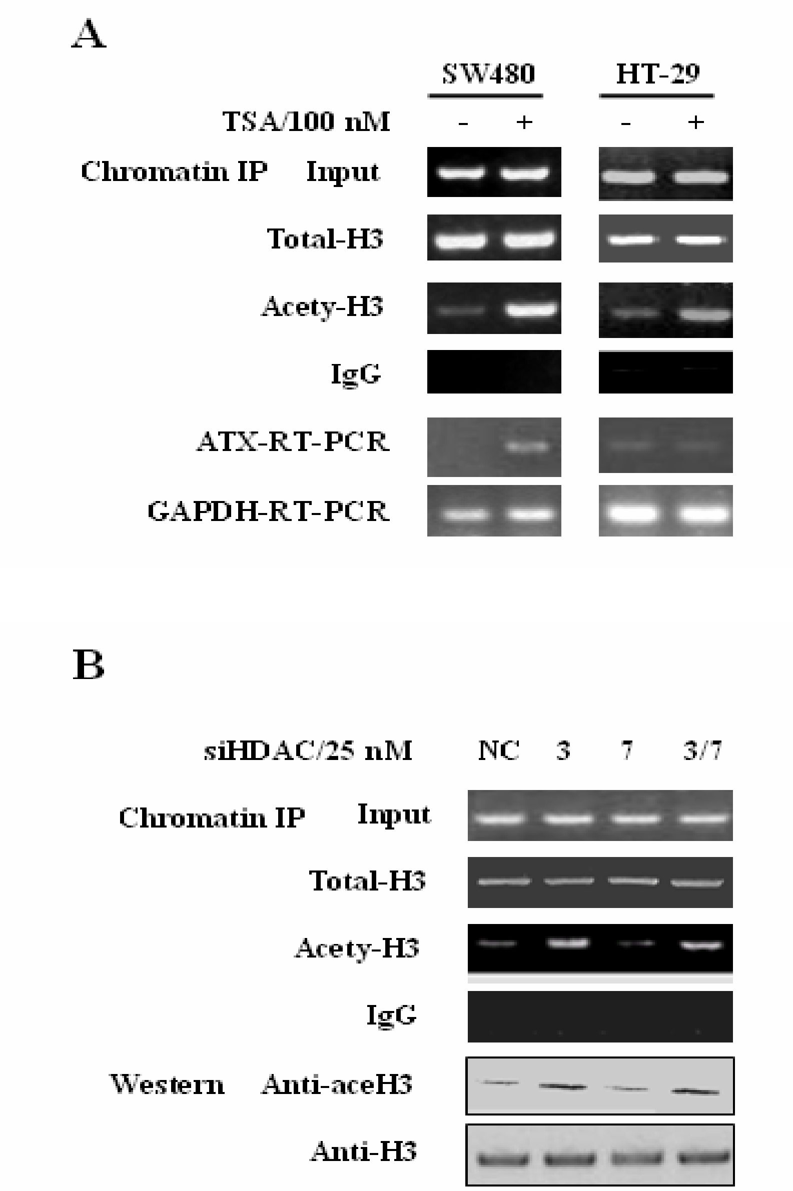

Supplement: Additional file 5 — figure S5 - Histone acetylation in ATX promoter region is insufficient to up-regulate ATX expression. A, SW480 and HT-29 cells were treated with or without TSA (100 nM) for 24 hrs, and then subjected to ChIP assays as described in Methods to detect the acetylated histone H3 in the ATX promoter region. ATX expression levels were detected by RT-PCR. B, SW480 cells were transfected with HDAC3 and/or HDAC7 siRNA(s) as indicated. The ChIP assays were performed 48 hrs post transfection to detect the acetylated histone H3 in ATX promoter region. The acetylation level of histone H3 (Lys9) in SW480 cell lysates was detected by Western blot analyses and normalized by the total histone H3. [file 1476-4598-10-18-S5.PDF]
