# Supplementary material for: Autotaxin is induced by TSA through HDAC3 and HDAC7 inhibition and antagonizes the TSA-induced cell apoptosis
Source: Mol Cancer. 2011 Feb 12;10:18. doi: 10.1186/1476-4598-10-18 (PMC3055229; doi:10.1186/1476-4598-10-18)
Supplement: Additional file 6 — figure S6 - Inhibition of ATX-LPA signaling enhanced TSA-induced apoptosis in serum-containing medium. The MDA-MB-231 cells were treated with TSA (1 μM) for 48 hrs in the conditional serum-free medium or serum (10%)-containing medium to detect the effect of serum on TSA-induced apoptosis. Furthermore, in serum (10%)-containing medium, MDA-MB-231 cells were pretreated with ATX inhibitor S32826 (1 μM) or LPA1/3 inhibitor Ki16425 (1 μM) for 1 hr, and then treated with TSA (1 μM) for 48 hrs. The cell apoptosis was measured after TSA treatment. The p value derived from Student's t test is (**) p < 0.001. [file 1476-4598-10-18-S6.PDF]

Supplementary figure 6– Inhibition of ATX-LPA signaling enhanced TSA-induced apoptosis in serum-containing medium.

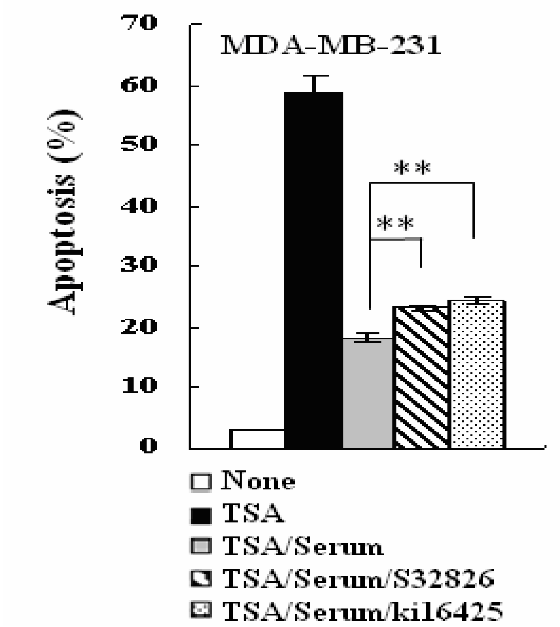

	None	TSA	TSA/serum	TSA/serum/s32826	TSA/serum/ki16425
Apoptosis (%)/ Mean ± SD	3.1 ± 0.1	58.9 ± 2.8	18.3 ± 0.7	23.2 ± 0.4	24.5 ± 0.6
